# How should we do equity, diversity and inclusion work in health professions education?

**DOI:** 10.12688/mep.19673.1

**Published:** 2023-06-08

**Authors:** Rashmi A. Kusurkar, Thirusha Naidu, Mohammed Ahmed Rashid

**Affiliations:** 1 Research in Education, Amsterdam UMC location Vrije Universiteit Amsterdam, De Boelelaan 1118, 1081HZ, The Netherlands; 2 Faculty of Psychology and Education, VU University Amsterdam, LEARN! Research Institute for Learning and Education, Amsterdam, The Netherlands; 3 Quality of Care, Amsterdam Public Health, Amsterdam, The Netherlands; 4 Department of Behavioural Medicine, Faculty of Health Sciences University of Kwa Zulu-Natal, Durban, South Africa; 5 Faculty of Medical Sciences, University College London, UCL Medical School, London, UK

**Keywords:** Equity, Diversity, Inclusion, Health Professions Education, Medical Education

## Abstract

This is an editorial for the special collection on equity, diversity and inclusion (EDI) for MedEdPublish. In this article, the guest advisors of this collection first reflect on the paradoxes in EDI in health professions education (HPE), then on the importance of recognising the existence of multiple authenticities on the basis of different contexts and settings, and finally encourage authors and readers to reflect on their position on the continuum of EDI work. They conclude the editorial by outlining the direction they wish to set for articles in the collection.

## Introduction

We welcome you to this special collection on equity, diversity and inclusion (EDI).

Rashmi Kusurkar is medical doctor trained in India, with a specialisation in physiology. Sixteen years ago, she moved to the Netherlands and completed her PhD in medical education. She currently works as a professor of inclusion and motivation in health professions education (HPE). Thirusha Naidu is a clinical psychologist and a woman and a 5 ^th^ generation South African descended from indentured slaves. She trained and practices in South Africa. Her work in medical education is rooted in her experiences of disparate healthcare and HPE. Mohammed Ahmed Rashid is a medical doctor who combines clinical work in an inner-city primary healthcare clinic with an academic role as professor of medical education and vice dean at University College London Medical School. His ancestors lived and worked under British colonial rule, and his research seeks to examine global power dynamics in medical education policy and practice.

Thus, all three guest advisors of this issue are first-, fifth- and second-generation migrants, respectively, in our current HPE contexts. Our views on EDI in HPE are shaped by our research as well as our first-hand experiences of working as migrants in our current contexts. We find this declaration of positionality crucial for readers to understand how we conceptualise and operationalise EDI, as well as how we are handling this special collection. In this editorial, we will first reflect on the paradoxes in EDI in HPE, then on the importance of recognising the existence of multiple authenticities on the basis of different contexts and settings, and finally encourage you to reflect on your position on the continuum of EDI work. The latter is key to how you read and understand the articles in this special collection. We will conclude the article by outlining the direction we wish to set for articles in this collection.

**Figure 1.**
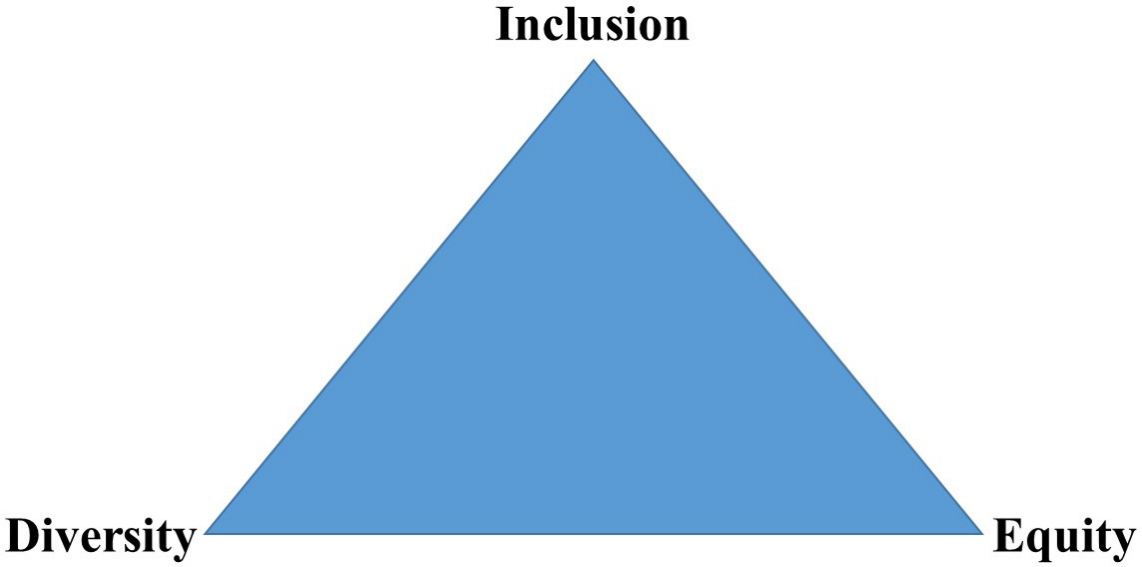
Equity, diversity and inclusion as an interrelated system.

Equity, diversity and inclusion in HPE should not be handled in isolation from each other, but as an interrelated system ( Rossi *et al.*, 2022). Acknowledging this, we approach EDI as a triad (see Figure 1).

We use the following conceptualizations of EDI:

*Equity* refers to the practice of embedding systems that ensure equal opportunities to all, regardless of their background or personal characteristics, with the aim of promotion of fairness, impartiality, and access. ( Annie. E. Casey Foundation; Kuper, 2016)

*Diversity* refers to representation of individuals of varied backgrounds in society in specific contexts such as professions, work organizations, research populations, etc.

*Inclusion* refers a sense of being included in a group or structure. Inclusion, through a true sense of belonging, empowers individuals to contribute authentically and meaningfully. ( Annie. E. Casey Foundation)

In this special collection, we will discuss how we can create equity and embrace the diversity of perspectives, rather than numbers, in order to achieve authentic inclusion for all individuals in HPE.

**Figure 2.**
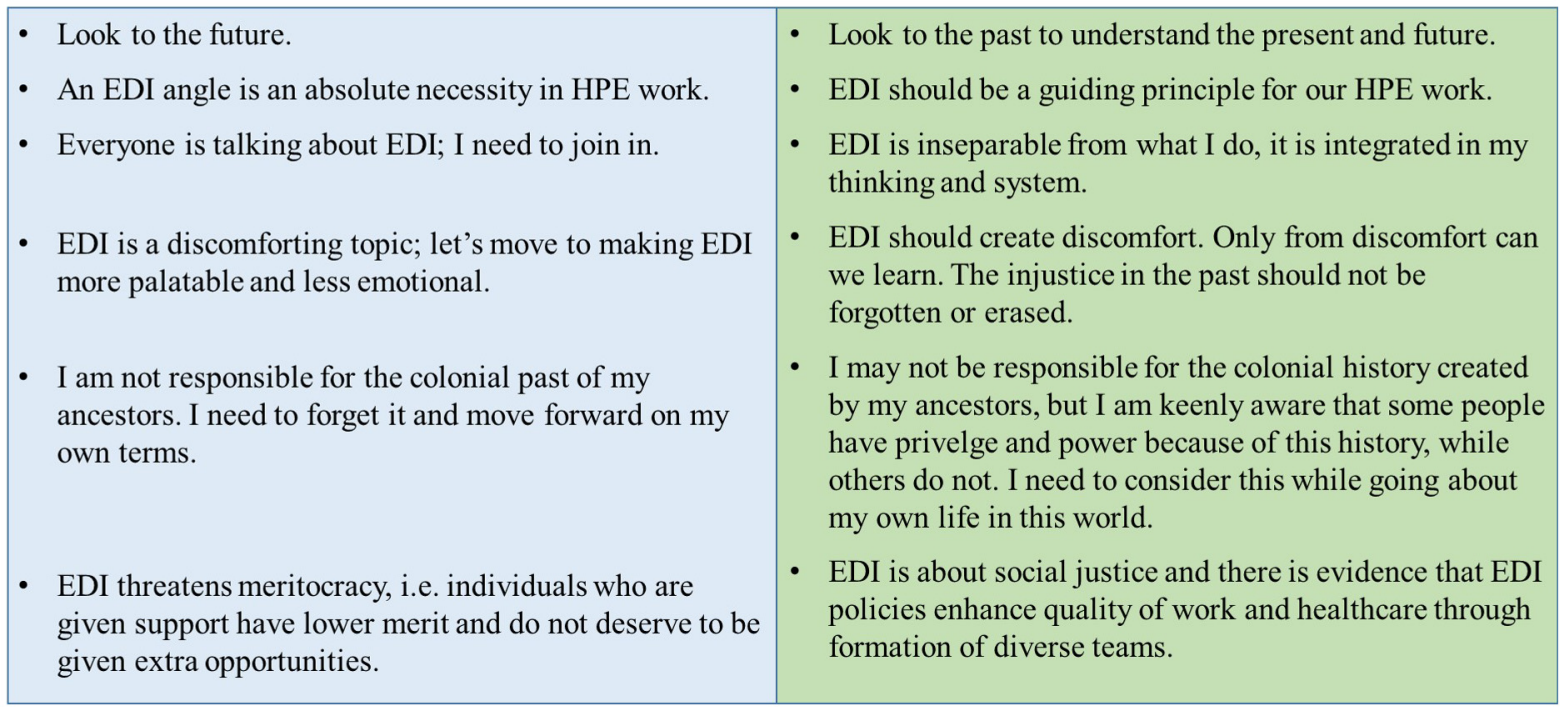
Paradoxes in EDI perspectives in HPE.

Even when diversity and equity are adequately approached, inclusion is not a given ( Rossi *et al.*, 2022). In contrast, inclusion needs to be actively encouraged, practised and role modelled. Driving EDI as a leader or proactively managing EDI as an individual teacher, educator or researcher is not easy, owing to the existing paradoxes in EDI perspectives (see Figure 2).

## The importance of historical perspectives in EDI work

In the opening paragraph of an article in which he reflects on lessons learnt from his 50 year career, HPE grandee Professor Ronald Harden reflects that “rather than revisiting the past, I am always more comfortable looking to the future” ( Harden, 2011). He is not alone in sharing this predilection for looking forward, rather than backward, as the field has long been dominated by ideas of ‘modernisation,’ ‘innovation,’ and ‘reform,’ all hinting at a need to keep one’s glance firmly ahead. Given that HPE students must be prepared for practice in the decades ahead, this forward gaze is rational and appropriate. Notwithstanding the logic behind this relentless attention given to the future, we believe that there is also much to be gained by pausing and looking backwards.

In their AMEE guide entitled ‘Looking back to move forward,’ Kuper *et al.* (2013) powerfully champion the importance of historical methodological research approaches in HPE, arguing that “history shows us that the structures of medical education are arbitrary and contingent”. Such approaches, therefore, enable the analysis of problems in the past and to see patterns that might otherwise have not been visible in the present. When considering the many current inequalities and injustices in our field, it is clear that these can be tracked back many years, decades, and sometimes even centuries, intertwined with many different scientific, medical, societal, and economic factors. Consider, for example, the many countries whose entire healthcare and higher education systems were thrust upon them by colonisers. As George Santayana said: *“Those who cannot remember the past are condemned to repeat it.”* We should not erase history, but face it and learn from it.

As Figure 2 demonstrates, EDI work in HPE is multi-faceted, complex, and oftentimes seemingly contradictory. As guest advisors, we acknowledge the inherent and compelling predisposition to focus forwards in a solution-oriented way, as well as the enormous value of uncovering and interrogating apparently ‘natural’ and inevitable current realities through historical analyses. We therefore welcome to this special issue articles that represent either (or indeed, both) of these different outlooks.

## Contextual authenticity

The notion that HPE should be ‘authentic’ has widely been accepted for many decades ( Herrington & Oliver, 2000; Hotchkiss *et al.*, 2002). We are likely, though, to have very different ideas of what is (or isn’t) authentic, according to our contexts. Envision, if you will, a historically prestigious but lately under-resourced public medical school in a bustling South Asian city, an impressively built new private medical school in an affluent Middle Eastern state, and a newly formed medical school focused on widening participation in an inner-city neighbourhood in North America. Having recently visited such schools and reflected on the endless differences in context between them, it is clear to us, as it would be to colleagues working in HPE, that each environment and setting requires a fundamentally different approach. It would be self-evident, for example, that each of these three schools would require a bespoke and carefully negotiated approach to EDI just as they would in other domains such as curriculum and assessment.

Calls for EDI work to be ‘authentic’ are valid, but informing the very concept of authenticity with contrasting meanings risks rendering it an empty word that is devoid of meaning. Efforts to find a single way to define authenticity are, we believe, not helpful. Rather, the concept of contextual authenticity legitimises multiple authenticities through recognising the central importance of responding to a host of local contextual factors.

EDI challenges inevitably differ across programmes, schools, cities, countries, and continents. It is therefore entirely appropriate that their explorations and analyses, along with any interventions that might be conceptualised or tested, should likewise differ. We acknowledge this multiplicity and encourage potential authors to situate their ideas and scholarship in the contexts in which they work and think. In particular, we recognise, encourage, and indeed celebrate the different methods and frameworks that we hope authors will bring to this collection.

## Spectrum of plurality in how the EDI movement is experienced

There is a spectrum or plurality to how we experience and respond to new and potentially threatening change. The advent of EDI and its practices and consequences may herald long overdue changes for some individuals and groups while unsettling the well-worn and expected trajectories of others. In this collection we invite those who find the EDI impetus baffling, unsettling or unnecessary to express their views, in addition to those who are convinced and confident of the need for EDI in HPE. Spotlighting one side of narrative may inhibit rich perspectives on the complexity inherent in EDI, and unreflective and poorly informed EDI applications. We would like to hear perspectives, experiences, debate, and discussion about how educators, scholars, clinicians, and students have actively resisted EDI through critique and visible activism. Perhaps you are observing with some discomfort the changes that EDI is bringing to your context but are hesitant about whether, or how, to resist the changes. What would you say if you were given the chance to speak without fear of reprisal? What are perspectives and concerns about EDI and how it is presented, implemented, and enacted?

The speed and pressure with which the wave of EDI change has seemed to sweep through all aspects of social life, including within HPE, may feel overwhelming for many. Some may be making the deliberate decision to disregard the changes that come with EDI in the belief that it is a passing trend. Are there factors or signals that suggest that attention on EDI may dwindle? Due to the attention on EDI you may accept, or have recently come to realize, that systems, structures and practices in HPE are discriminatory against marginalized persons, however you are not certain that current EDI activists are approaching the challenges in respectful or productive ways. We would like to hear about this as well as the potential solutions you would offer. Are there gains that we are potentially losing as a field amid the EDI wave?

## Why do we want to give a place to both ‘for’ and ‘against’ perspectives in this special collection?

We believe that only when researchers and educators take supporting perspectives as well as reservations against EDI on board, will they bring about an impact in the larger society where these perspectives are mirrored. It’s time to have an open discussion about why or why not EDI, for whom and for whom not, who does or does not benefit from EDI measures, who suffers or does not suffer from EDI measures, and what are the possible long-term effects. On conclusion of this collection, we will contribute a reflection of all the voices in it. We hope to receive theoretical, empirical, opinion, case study, review, and practical papers on topics such as (but not excluding others):

- EDI work: A blessing or a curse/justice or injustice
- An EDI perspective on different topics in HPE, such as admissions/selection, identity development, competency development and performance, curricula, experiences in education, patient outcomes, etc.
- EDI as a guiding principle/or not in HPE
- EDI in HPE, health literature and publishing practices
- The EDI versus meritocracy debate: Are they mutually exclusive?
- EDI at local, national, regional, or global levels

We look forward to your boundary-pushing contributions!
